# Long Non-Coding RNA LINC00355 Promotes the Development and Progression of Colorectal Cancer by Elevating Guanine Nucleotide Exchange Factor T Expression *via* RNA Binding Protein lin-28 Homolog A

**DOI:** 10.3389/fonc.2020.582669

**Published:** 2020-12-14

**Authors:** Yuanyuan Wang, Bing Zhang, Ge Gao, Yinping Zhang, Qingxin Xia

**Affiliations:** Department of Pathology, Affiliated Cancer Hospital of Zhengzhou University, Henan Cancer Hospital, Zhengzhou, China

**Keywords:** progress, colorectal cancer, LIN28A, LINC00355, GEFT

## Abstract

**Background:**

Our previous study showed that guanine nucleotide exchange factor T (GEFT) was highly expressed in colorectal cancer (CRC) tissues and CRC patients with high GEFT expression had a poor prognosis, and suggested the close link of GEFT expression and CRC tumorigenesis/metastasis. In this text, the roles and upstream regulatory mechanisms of GEFT in the development and progression of CRC were further investigated.

**Methods:**

Expression levels of GEFT mRNA and LINC00355 was measured by RT-qPCR assay. Protein levels of lin-28 homologue A (LIN28A) and GEFT were determined by western blot assay. Cell proliferative, migratory, and invasive capacities were assessed by CCK-8, Transwell migration and invasion assays, respectively. The effect of GEFT knockdown on CRC tumorigenesis was examined by mouse xenograft experiments *in vivo*. GEFT mRNA stability was examined by actinomycin D assay. The relationships of LINC000355, LIN28A, and GEFT were explored by RNA pull down and RIP assays.

**Results:**

GEFT was highly expressed in CRC tissues and cell lines. GEFT knockdown inhibited CRC cell proliferation, migration, and invasion, and hindered CRC xenograft tumor growth. GEFT overexpression alleviated the detrimental effects of LINC00355 loss on CRC cell proliferation, migration, and invasion. LINC00355 promoted GEFT expression and enhanced GEFT mRNA stability *via* LIN28A. LIN28A knockdown weakened the promotive effect of LINC00355 on CRC cell proliferation, migration, and invasion.

**Conclusion:**

LINC00355 facilitated CRC tumorigenesis and progression by increasing GEFT expression *via* LIN28A, deepening our understanding on roles and upstream regulatory mechanisms of GEFT in CRC development and progression.

## Introduction

Colorectal cancer (CRC) is responsible for approximately 10% of all diagnosed malignancy cases and 8.9% of all cancer-related deaths worldwide (1). The incidence and mortality rates of CRC rank the third and the fourth globally, respectively (1). CRC is often diagnosed at the advanced stage (2). Patients with advanced CRC have a poor prognosis with the 5-year cumulative survival of 14–15% (1). An in-depth understanding on molecular biology of CRC might contribute to the better management of CRC.

Guanine nucleotide exchange factor T (GEFT), also named as Rho guanine nucleotide exchange factor 25 (ARHGEF25) and p63RhoGEF, can stimulate the activation of RhoA, Rac1, and Cdc42 GTPases in different cells (3–5), which have been found to be involved in the regulation of multiple pathways and biological processes such as proliferation, migration, and invasion (6, 7). In addition, previous studies showed that GEFT functioned as a potential oncogene in some malignancies such as breast cancer (8) and rhabdomyosarcoma (RMS) (9). For instance, GEFT overexpression improved the proliferative, migratory, and invasive capacities of RMS cells, inhibited RMS cell apoptosis, and promoted RMS xenograft tumor growth and lung metastasis through activating Rac1/Cdc42-PAK1 pathways (10). Our prior study showed that GEFT expression was notably increased in CRC tissues than that in normal intestinal mucosa and CRC patients with high GEFT expression had a poor prognosis (11). Moreover, GEFT expression was associated with lymph node metastasis and vessel carcinoma embolus in CRC (11). In addition, a negative correlation was observed between GEFT and mismatch repair protein expression in CRC (11). These data suggested the close link of GEFT and CRC tumorigenesis/metastasis. However, the roles and molecular basis of GEFT in the development and progression of CRC were poorly defined.

It is well known that the regulation of gene expression and function can be mediated by plenty of RNA binding proteins (RBPs), which have the binding activity with diverse RNAs including coding and non-coding RNAs (12, 13). RBPs have been identified as crucial players in controlling RNAs’ metabolism and lifecycle, including RNA biogenesis, maturation, translation, stability, and degradation (12, 13). The dysregulation of RBPs was closely linked with the pathogenesis of numerous diseases including cancers (14, 15). For example, RBP RNA binding motif protein 38 (RBM38) inhibited cell proliferation through enhancing PTEN mRNA stability in breast cancer (16). Long non-coding RNAs (lncRNAs), a group of transcripts (longer than 200 nucleotides in length) with little or no protein-coding potential, can regulate gene expression at transcriptional and post-transcriptional levels (17, 18). Moreover, lncRNAs usually exert their functions through binding with other molecules including RBPs in various physiological and pathological processes such as cancer initiation and progression (17, 19). For example, lncRNA cancer susceptibility 9 (CASC9) knockdown suppressed hepatocellular carcinoma (HCC) cell proliferation and induced cell apoptosis *in vitro* and hampered HCC xenograft tumor growth *in vivo* by binding with RBP heterogeneous nuclear ribonucleoprotein L (HNRNPL), where the CASC9-HNRNPL complex could co-regulate the expression of genes associated with AKT signaling pathway (20). LncRNA PDCD4-AS1 enhanced the stability of PDCD4 mRNA through reducing binding activity of RBP HuR with PDCD4 mRNA 3’UTR (21).

In this text, the effects of GEFT knockdown on proliferation, migration, and invasion of CRC cells and CRC xenograft tumor growth were examined. Also, lncRNAs and related RBPs that could regulate GEFT expression and functions in CRC were further delved.

## Materials and Methods

### Clinical Samples

CRC tumor tissues and adjacent normal tissues were collected from 60 patients with CRC who underwent surgical resection. The detailed information of clinical samples was presented in our previous article (11) and **Table 1**. Our study was performed with the approval of Institutional Ethics Committee of our hospital and the written informed consents from all patients.

**Table 1:** Association between LINCOO355 expression and clinicopathologic features in patients with colorectal cancer.

Variable	Case	Relative LINCOO355 level		P-value
		High (%)	Low (%)	
Sex				0.8864
Male	33	15 (45.45)	18 (54.55)	
Female	27	12 (44.44)	15 (55.56)	
Age				0.4374
≥60	34	16 (47.06)	18 (52.94)	
<60	26	11 (42.31)	15 (57.69)	
Tumor diameter				0.0319*
≥5cm	40	20 (50.00)	20 (50.00)	
<5cm	20	7 (35.00)	13 (65.00)	
Location				0.7739
Right hemicolon	12	5 (41.67)	7 (58.33)	
Sigmoid colon	15	7 (46.67)	8 (53.33)	
Rectum	33	15 (45.45)	18 (54.55)	
Tumor differentiation				0.2564
Moderate/well	52	23 (42.23)	29 (55.77)	
Poor	8	4 (50.00)	4 (50.00)	
Mucinous adenocarcinoma				0.7752
Yes	7	3 (42.86)	4 (57.14)	
No	53	24 (45.28)	29 (54.72)	
Depth of invasion				0.6695
T3	14	6 (42.86)	8 (57.14)	
T4	46	21 (45.65)	25 (54.35)	
Nerve invasion				0.0601
Yes	3	1 (33.33)	2 (66.67)	
No	57	26 (45.61)	31 (54.39)	
Vessel carcinoma embolus				0.0156*
Yes	32	17 (53.13)	15 (46.87)	
No	28	10 (35.71)	18 (64.29)	
TNM stage				0.1552
I and II	32	16 (50.00)	16 (50.00)	
III and IV	28	11 (39.29)	17 (60.71)	
Lymph node metastasis				0.0230*
Yes	30	16 (53.33)	14 (46.67)	
No	30	11 (36.67)	19 (63.33)	
Distant metastasis				0.0896
Yes	11	6 (54.54)	5 (45.46)	
No	49	21 (42.86)	28 (57.14)	

*P < 0.05.

### Cell Culture

CRC cell lines (SW480, HT-29, and HCT-116) and a normal human colon epithelial cell line (FHC) were purchased from American Type Culture Collection (ATCC, Manassas, VA, USA). SW480 cells were cultured in Leibovitz’s L-15 medium (Thermo Scientific, Rockford, IL, USA) supplemented with 10% fetal bovine serum (FBS, Thermo Scientific) at 37°C in a 100% air atmosphere. HT-29 and HCT-116 cells were grown in McCoy's 5A (Modified) medium (Thermo Scientific) containing 10% FBS at 37°C in a 95% air / 5% CO_2_ atmosphere. FHC cells were cultured in DMEM/F12 medium (Thermo Scientific) containing 25 mM HEPES (Sigma-Aldrich, St. Louis, MO, USA), 10 ng/ml cholera toxin (MedChemExpress, MJ, NJ, USA), 0.005 mg/ml insulin (Sigma-Aldrich), 0.005 mg/ml transferrin (Sigma-Aldrich), 100 ng/ml hydrocortisone (Sigma-Aldrich), 20 ng/ml human recombinant EGF (Sigma-Aldrich), and 10% FBS (Thermo Scientific) at 37°C in a 95% air / 5% CO_2_ atmosphere.

### Reagents and Cell Transfection

Small interference RNAs (siRNAs) targeting GEFT, LIN28A, LINC00355 and the negative control were synthesized by GenePharma Co., Ltd. (Shanghai, China). GEFT, LIN28A, and LINC00355 overexpression plasmids were customized from Sangon Biotech Co., Ltd. (Shanghai, China). Cell transfection was conducted using Lipofectamine 3000 reagent (Thermo Scientific) following the protocols of manufacturer. SiRNA target sequences were as follows: 5’-CGTGATGGTTGATAGCTAA-3’for LIN28A#1; 5’-GGGTTGTGATGACAGGCAA-3’ LIN28A#2; 5’-GGCTCAGCTATTCATCAAA-3’ for GEFT#1; 5’-CCGAGACTATTTCTTGCAA-3’ for GEFT#2; 5’-CCTCTCTGTTGAGAGCTAA-3’ for LINC00355#1; 5’-GGACTGTAAACTAGTTCAA-3’ for LINC00355#2.

### RNA Extraction and Reverse Transcription-Quantitative PCR Assay

Total RNA was extracted from CRC tissues, normal tissues, CRC cell lines, and FHC using Trizol reagent (Thermo Scientific). cDNA was synthesized using M-MLV Reverse Transcriptase (Thermo Scientific) and subsequent real-time quantitative PCR reactions were performed using SYBR™ Green PCR Master Mix (Thermo Scientific) and specific primers on ABI 7500 Real-Time PCR System (Applied Biosystems, Grand Island, NY, USA). GAPDH functioned as the housekeeping gene to normalize the expression of LINC00355, MIR31HG, and GEFT. Quantitative PCR primers were presented as follows: 5’-CCAACAGGAAGCAAGCACAG-3’ (forward) and 5’-CTAACACTTTGGGCAGCGTTT-3’ (reverse) for LINC00355, 5’-CTCAAGGCCAGTGTAGAGCC-3’ (forward) and 5’-TGCTGCATGGAACATGACCT-3’ (reverse) for MIR31HG, 5’-CCCAAGTCAGAGCATGTGGT-3’ (forward) and 5’-CCCTCAAATCCCCGCAATCT-3’ (reverse) for GEFT, 5’-TTGCCCTCAACGACCACTTT-3’ (forward) and 5’-TGGTCCAGGGGTCTTACTCC-3’ (reverse) for GAPDH.

### GEFT mRNA Stability Assay

At 24 h after transfection, actinomycin D (ActD, 5 µg/ml, Sigma-Aldrich Inc.) was added into media of transfected cells. At the indicated time points (0, 2, 4, 6 h) post ActD addition, RNA was extracted and GEFT mRNA level was determined by RT-qPCR assay.

### Western Blot Assay

Western blot assay was performed using the standard experimental procedures. Proteins (30 μg) were separated by 10% sodium dodecyl sulfate-polyacrylamide gel electrophoresis (SDS-PAGE). Primary antibodies against LIN28A (1:5,000 dilution, ab124765, Abcam, Cambridge, UK), GEFT (1:1,000 dilution, ab127690, Abcam), GAPDH (1:5,000 dilution, ab181602, Abcam), and goat-anti-rabbit secondary antibody conjugated with horseradish peroxidase (1:5,000 dilution, ab205718, Abcam) were used in the western blot assay. Protein signals were detected using the Pierce™ ECL Western Blotting Substrate (Thermo Scientific).

### Luciferase Reporter Assay

Wild type GEFT 3’UTR region was cloned into pGL3-Basic firefly luciferase vector by Hanbio Biotechnology Co., Ltd. (Shanghai, China), and the recombinant plasmid was named as WT GEFT 3’UTR reporter. Also, mutant (MUT) GEFT 3’UTR reporter carrying the mutant binding motif was also constructed by Hanbio Biotechnology Co., Ltd. HCT-116 cells transfected with pcDNA3.1 empty vector or LIN28A overexpression plasmid were co-transfected with pRL-TK Renilla luciferase plasmid, pGL3-Basic luciferase vector/construct. At 48 h post transfection, luciferase activities were measured by Dual-Luciferase Reporter Assay (Promega, Madison, WI, USA) following the protocols of manufacturer.

### Cell Proliferative Ability Analysis

Cell proliferative ability was assessed at the indicated time points after transfection using the Cell Counting Kit-8 (CCK-8) assay kit (Dojindo Molecular Technologies, Rockville, MD, USA) according to the instructions of manufacturer.

### Transwell Migration and Invasion Assay

Cell migratory and invasive abilities were measured using the 24-well Transwell insert chambers (Corning Inc. New York, NY, USA) with the filtration membrane pore size of 8 µm. Prior to cell invasion assays, the membranes were coated with Matrigel (BD Bioscience, San Diego, CA, USA). Transfected cells in serum-free medium were seeded in the upper chambers and medium supplemented with 10% FBS was added to the lower chambers. After 24 h of incubation, non-migrated or non-invaded cells were removed using a cotton swab and migratory or invasive cells were fixed, stained, imaged, and counted under a light microscope.

### RNA Immunoprecipitation Assay

The binding potential between LIN28A and GEFT/LINC00355 in transfected or un-transfected HCT-116 cells was measured through RIP assay at 48 h post transfection using the Magna RIP RNA-Binding Protein Immunoprecipitation Kit (Millipore, Temecula, CA, USA) together with IgG (ab172730, 1:20 dilution, Abcam) or LIN28A antibody (ab124765, 1:50 dilution, Abcam) according to the instructions of manufacturer. LINC00355 or GEFT mRNA level enriched by IgG or LIN28A antibody were determined by RT-qPCR assay.

### RNA Pull-Down Assay

Biotin-labeled sense or antisense GEFT 3’UTR, biotinylated sense or antisense LINC00355 were customized from Sangon Biotech Co., Ltd. (Shanghai, China). RNA pull-down assay was carried out in whole-cell lysates of HCT-116 cells using the Pierce™ Magnetic RNA-Protein Pull-Down Kit (Thermo Scientific) referring to the instructions of manufacturer. LIN28A protein level pulled down by biotin-labeled transcripts was examined by western blot assay.

### Animal Experiments

The oligos for sh-GEFT were: 5’-CCGGAAGGCTCAGCTATTCATCAAACTCGAGTTTGATGAATAGCTGAGCCTTTTTTTG-3’ (forward oligo) and 5’-AATTCAAAAAAAGGCTCAGCTATTCATCAAACTCGAG TTTGATGAATAGCTGAGCCTT-3’ (reverse oligo). The sh-GEFT oligos were constructed into pLKO.1 vector. Lentiviruses expressing shRNAs against GEFT (sh-GEFT) and control lentiviruses (sh-con) were obtained from Hanbio Biotechnology Co., Ltd. (Shanghai, China). HCT-116 cells were infected with sh-con or sh-GEFT lentiviruses. At 72 h after lentivirus infection, cells were screened out for 7 days using 1 μg/ml puromycin to establish stably transfected cell lines with or without GEFT knockdown.

Our animal experiments were approved by the Animal Care and Use Committee of our hospital and performed with the standard experimental procedures. BALB/c nude mice (n = 18, 8 weeks old) were purchased from Laboratory Animal Center of Zhengzhou University (Zhengzhou, China) and raised for 1 week under standard conditions to allow them to adapt to the environment. Mice were randomly divided into sham, sh-con, or sh-GEFT group with six mice in each group.

Normal HCT-116 cells (2 × 10^6^), HCT-116 cells infected with sh-con lentiviruses (2 × 10^6^), or HCT-116 cells infected with sh-GEFT lentiviruses (2 × 10^6^) were subcutaneously inoculated into the left hip areas of mice in sham, sh-con, or sh-GEFT group, respectively. Sham group referred to xenograft tumors derived from normal HCT-116 cells. The sh-con or sh-GEFT group represented xenograft tumors derived from HCT-116 cells infected with sh-con or sh-GEFT lentiviruses, respectively. Tumor volume was monitored using calipers and calculated using the formula: V = 0.50 × length × width^2^, where length represents longest tumor diameter and width represents the corresponding perpendicular diameter. Tumors were resected and weighed on day 25 after cell injection.

### Statistical Analysis

Data were analyzed using GraphPad Prism software 5.0 (La Jolla, CA, USA) and expressed as mean ± standard deviation. Difference of two group data was analyzed using Student’s *t*-test. Difference of more than two group data was analyzed using one-way analysis of variance (ANOVA) and Tukey’s *post-hoc* test. *P* < 0.05 represents that difference was statistically significant.

## Results

### Guanine Nucleotide Exchange Factor T Was Highly Expressed in Colorectal Cancer Tissues and Cell Lines

**Figure 1:**
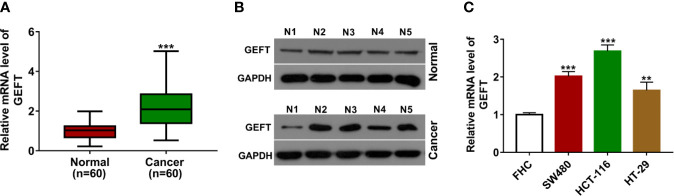
GEFT was highly expressed in CRC tissues and cell lines. **(A)** GEFT mRNA level was measured by RT-qPCR assay in 60 pairs of CRC tissues and adjacent normal tissues. **(B)** GEFT protein level was examined by western blot assay in five pairs of random CRC tissues and adjacent normal tissues. **(C)** GEFT mRNA level was determined by RT-qPCR assay in FHC, SW480, HCT-116, and HT-29 cells. **P < 0.01. ***P < 0.001.

Firstly, RT-qPCR assay showed that GEFT mRNA level was notably increased in CRC tumor tissues (n = 60) compared to adjacent normal tissues (n = 60) (**Figure 1A**). Western blot assay further demonstrated that GEFT protein level was markedly increased in five random CRC tumor tissues relative to corresponding normal tissues (**Figure 1B**). Also, a remarkable up-regulation of GEFT mRNA level was observed in multiple CRC cell lines (SW480, HCT-116, and HT-29) than that in a normal human colon epithelial cell line (FHC) (**Figure 1C**).

### Guanine Nucleotide Exchange Factor T Knockdown Inhibited Colorectal Cancer Cell Proliferation, Migration, and Invasion *In Vitro* and Hampered Colorectal Cancer Xenograft Tumor Growth *In Vivo*

**Figure 2:**
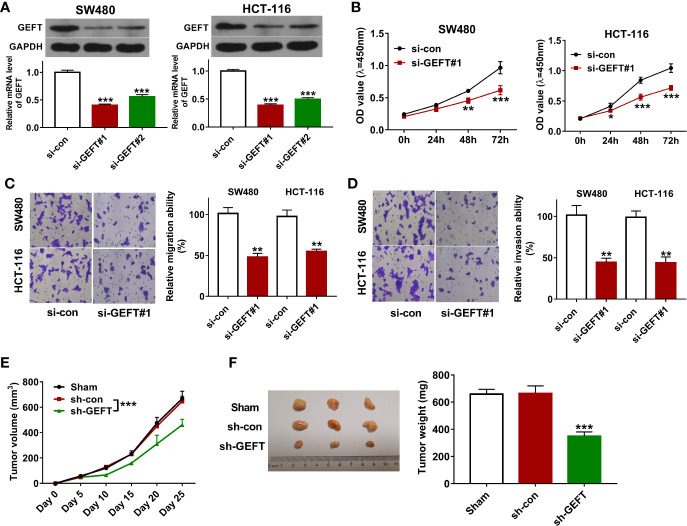
GEFT knockdown inhibited CRC cell proliferation, migration, and invasion *in vitro* and hampered CRC xenograft tumor growth *in vivo*. **(A)** SW480 and HCT-116 cells were transfected with si-con, si-GEFT#1, or si-GEFT#2. Forty-eight hours later, GEFT mRNA and protein levels were measured by RT-qPCR and western blot assays, respectively. **(B–D)** SW480 and HCT-116 cells were transfected with si-con or si-GEFT#1. **(B)** Cell proliferative ability was assessed by CCK-8 assay at 0, 24, 48, 72 h post transfection. **(C, D)** At 24 h post transfection, cell migratory and invasive potential was estimated by Transwell migration and invasion assays, respectively. **(E)** Tumor volume was monitored at the indicated time points post CRC cell injection. **(F)** Tumors were resected and weighed on day 25 post CRC cell inoculation. *P < 0.05. **P < 0.01. ***P < 0.001.

To further investigate the function of GEFT in CRC tumorigenesis and progression, two siRNAs targeting GEFT (si-GEFT#1, si-GEFT#2) and a scramble control siRNA (si-con) were designed and synthesized. Knockdown efficiency analysis revealed that the transfection of si-GEFT#1 or si-GEFT#2 led to notable reduction of GEFT expression in SW480 and HCT-116 cells compared with si-con group (**Figure 2A**). Considering higher knockdown efficiency of si-GEFT#1 *versus* si-GEFT#2, si-GEFT#1 was used for following loss-of-function experiments. CCK-8 assay showed that GEFT loss led to the noticeable down-regulation of cell proliferative ability in SW480 and HCT-116 cells (**Figure 2B**). Also, Transwell migration and invasion assays revealed that GEFT knockdown remarkably weakened the migratory and invasive potential of SW480 and HCT-116 cells (**Figures 2C, D**). *In vivo* xenograft tumor experiments further demonstrated that GEFT knockdown hindered CRC tumor growth (**Figures 2E, F**).

### LINC00355 Promoted Guanine Nucleotide Exchange Factor T Expression and Enhanced Guanine Nucleotide Exchange Factor T mRNA Stability

**Figure 3:**
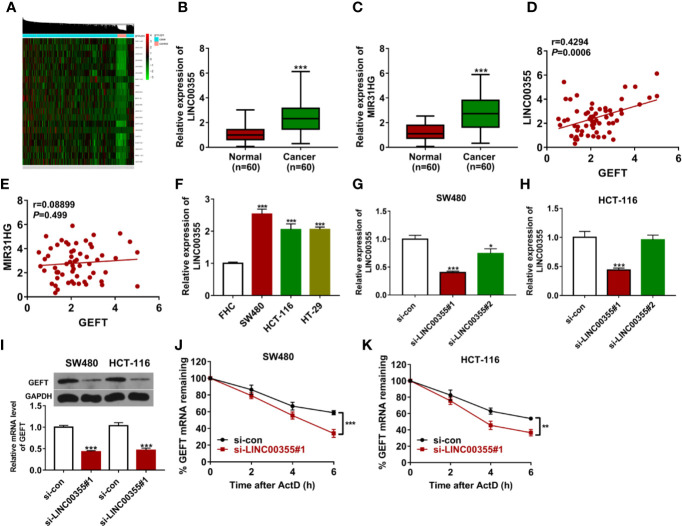
LINC00355 promoted GEFT expression and enhanced GEFT mRNA stability. **(A)** Heat map of 20 interested lncRNAs in CRC tumor tissues versus normal tissues. **(B, C)** Expression levels of LINC00355 and MIR31HG in 60 pairs of CRC tumor tissues and adjacent normal tissues were measured by RT-qPCR assay. **(D, E)** Correlation analysis of GEFT and MIR31HG or LINC00355 expression in 60 cases of CRC tumor tissues. **(F)** Expression analysis of LINC00355 in FHC and several CRC cell lines. **(G, H)** SW480 and HCT-116 cells were transfected with si-con, si-LINC00355#1, or si-LINC00355#2. At 48 h post transfection, LINC00355 level was measured by RT-qPCR assay. **(I–K)** SW480 and HCT-116 cells were transfected with si-con or si-LINC00355#1. **(I)** At 48 h after transfection, GEFT mRNA and protein levels were determined by RT-qPCR and western blot assays, respectively. **(J, K)** The effect of LINC00355 knockdown on GEFT mRNA stability was assessed by actinomycin D assay. **P < 0.01. ***P < 0.001.

Considering the crucial regulatory roles of lncRNAs in gene expression and function, expression profiles of lncRNAs in CRC tumor tissues (n = 473) *versus* adjacent normal tissues (n = 42) were downloaded from The Cancer Genome Atlas (TCGA) database. Next, differentially expressed lncRNAs in CRC tumor tissues versus adjacent normal tissues were identified. Among the top 100 up-regulated lncRNAs (**Supplementary Excel**), 20 lncRNAs implicated in cancer progression were screened out (**Figure 3A**, **Supplementary Table 1**). Among these 20 lncRNAs, 6 lncRNAs [*i.e.* FEZF1-AS1 (22, 23), LINC01234 (24, 25), LINC00460 (26–29), LINC00659 (30), MIR31HG (31), and LINC00355 (32)] have been documented to be closely linked with CRC progression and the prognosis of CRC patients. In addition, the roles or/and molecular mechanisms of FEZF1-AS1, LINC01234, LINC00460, and LINC00659 in the development of CRC have been examined in previous studies and few studies have been conducted to explore the functions and molecular basis of MIR31HG and LINC00355 in CRC progression. Hence, MIR31HG and LINC00355 were selected for further study. Expression analysis revealed that LINC00355 and MIR31HG expression was significantly up-regulated in CRC tumor tissues than that in adjacent normal tissues (**Figures 3B, C**). Correlation analysis presented that LINC00355 expression was positively associated with GEFT expression in CRC tumor tissues (n = 60) (**Figure 3D**). However, there was no obvious correlation between GEFT and MIR31HG expression in CRC tumor tissues (**Figure 3E**). Consequently, the regulatory effect of LINC00355 on GEFT was further explored. RT-qPCR assay further validated that LINC00355 expression was notably up-regulated in several CRC cell lines than that in FHC cell line (**Figure 3F**). Transfection efficiency analysis showed that the introduction of si-LINC00355#1 triggered the noticeable down-regulation of LINC00355 level in SW480 and HCT-116 cells relative to si-con group (**Figures 3G, H**). RT-qPCR and western blot assays demonstrated that LINC00355 knockdown inhibited GEFT mRNA and protein expression in SW480 and HCT-116 cells (**Figure 3I**). The down-regulation of mRNA level might be caused by the reduction of mRNA synthesis capacity or improvement of mRNA degradation activity. Hence, the effect of LINC00355 loss on GEFT mRNA stability was tested by actinomycin D assay. Results showed that LINC00355 knockdown could notably reduce GEFT mRNA stability in SW480 and HCT-116 cells (**Figures 3J, K**).

### Guanine Nucleotide Exchange Factor T Overexpression Lessened the Detrimental Effects of LINC00355 Loss on Colorectal Cancer Cell Proliferation, Migration, and Invasion

**Figure 4:**
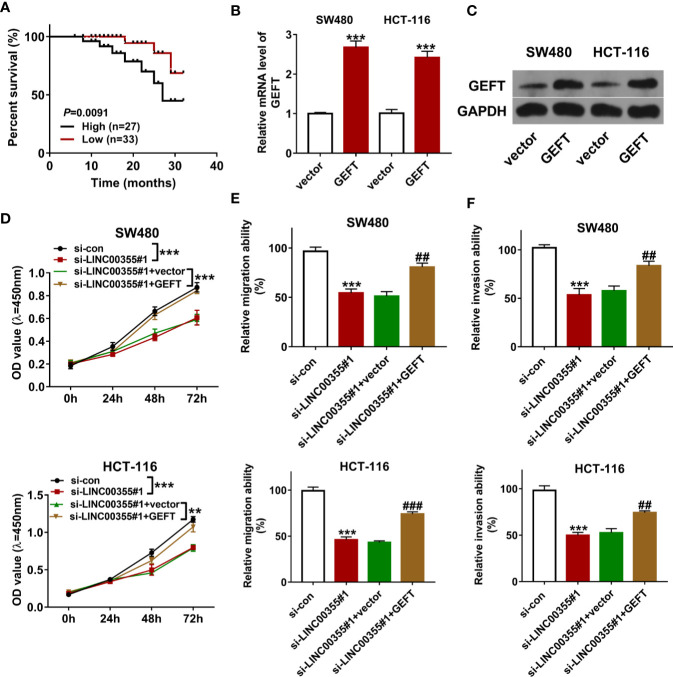
GEFT overexpression lessened the detrimental effects of LINC00355 loss on CRC cell proliferation, migration, and invasion. **(A)** Kaplan-Meier survival analysis for CRC patients based on the difference of LINC00355 expression. **(B, C)** SW480 and HCT-116 cells were transfected with GEFT overexpression plasmid or empty vector, followed by GEFT mRNA and protein expression levels at 48 h post transfection. **(D–F)** SW480 and HCT-116 cells were transfected with si-con, si- LINC00355#1, si-LINC00355#1 + vector, or si-LINC00355#1 + GEFT. **(D)** Cell proliferative ability was assessed by CCK-8 assay at 0, 24, 48, or 72 h post transfection. **(E, F)** At 24 h after transfection, cell migratory and invasive abilities were estimated by Transwell assays, respectively. **P < 0.01. ***P < 0.001. ^##^ *P* < 0.01. ^###^ *P* < 0.001.

Next, CRC tumor tissues were divided into LINC00355 high expression group (≥ mean value) and low expression group (<mean value) with the mean value of LINC00355 expression in CRC tumor tissues as the cutoff point. Survival analysis revealed that CRC patients with high LINC00355 expression had a poor overall survival (**Figure 4A**). Moreover, clinical analysis revealed that LINC00355 expression was associated with tumor size, vessel carcinoma embolus, and lymph node metastasis in CRC (**Table 1**). Transfection efficiency analysis revealed that the transfection of GEFT overexpression plasmid led to the remarkable up-regulation of GEFT expression (**Figures 4B, C**). Functional analysis showed that LINC00355 knockdown notably weakened the proliferative, migratory, and invasive abilities of SW480 and HCT-116 cells (**Figures 4D–F**). Enforced expression of GEFT alleviated the detrimental effects of LINC00355 loss on cell proliferation, migration, and invasion in SW480 and HCT-116 cells (**Figures 4D–F**).

### LINC00355 Promoted Guanine Nucleotide Exchange Factor T Expression and Improved Guanine Nucleotide Exchange Factor T mRNA Stability by LIN28A

**Figure 5:**
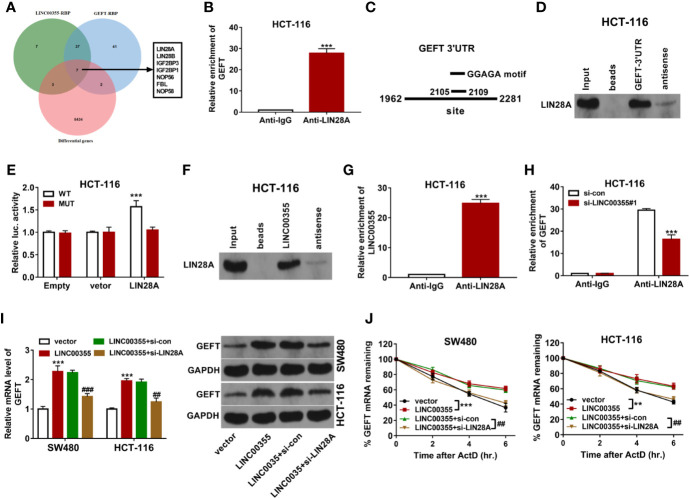
LINC00355 promoted GEFT expression and improved GEFT mRNA stability by recruiting RBP LIN28A. **(A)** Screening of seven CRC differentially expressed RBPs that have the potential to interact with both LINC00355 and GEFT through Venn analysis. Green: Starbase-predicted RBPs with the possibility to interact with LINC00355; blue: Starbase-predicted RBPs with the probability to interact with GEFT; red: Differentially expressed genes in CRC tissues *versus* normal tissues. **(B)** GEFT mRNA level enriched by IgG or LIN28A antibody were examined by RIP coupled with RT-qPCR assay in HCT-116 cells. **(C)** Predicted binding sites between LIN28A and GEFT 3’UTR. **(D)** RNA pull-down and western blot assays were performed to test LIN28A protein level pulled down by biotinylated sense or antisense GEFT 3’UTR. **(E)** HCT-116 cells transfected with pcDNA3.1 empty vector or LIN28A overexpression plasmid were co-transfected with pRL-TK Renilla luciferase plasmid and pGL3-Basic luciferase vector/construct, followed by the measurement of luciferase activity at 48 h post transfection. **(F)** RNA pull-down and western blot assays were performed to test LIN28A protein level pulled down by biotinylated sense or antisense LINC00355. **(G)** LINC00355 level enriched by IgG or LIN28A antibody was examined by RIP coupled with RT-qPCR assay. **(H)** HCT-116 cells were transfected with si-con or si- LINC00355#1. At 48 h post transfection, RIP coupled with RT-qPCR assay was performed to test GEFT mRNA level enriched by IgG or LIN28A antibody. **(I, J)** SW480 and HCT-116 cells were transfected with empty vector, LINC00355 overexpression plasmid, LINC00355 overexpression plasmid + si-con, LINC00355 overexpression plasmid + si-LIN28A. **(I)** At 48 h upon transfection, GEFT mRNA and protein levels were measured by RT-qPCR and western blot assays, respectively. **(J)** At 24 h post transfection, cells were treated with actinomycin D. At the indicated time points after actinomycin D treatment, GEFT mRNA level was measured by RT-qPCR assay. **P < 0.01. ***P < 0.001. ^##^ *P* < 0.01. ^###^ *P* < 0.001.

Next, RBPs that had the likelihood to interact with both LINC00355 and GEFT were searched by Starbase prediction analysis. Venn analysis revealed that 44 RBPs had the possibility to interact with both LINC00355 and GEFT (**Figure 5A**). Also, expression profiles of mRNAs in CRC tumor tissues (n = 473) and adjacent normal tissues (n = 42) were downloaded from The Cancer Genome Atlas (TCGA) database, followed by the differential expression analysis of genes in CRC tissues *versus* normal tissues. Combined with the gene differential expression data, seven RBPs were found to be differentially expressed in CRC versus normal tissues among these 44 common RBPs (**Supplementary Table 2**, **Figure 5A**). Next, the associations of these seven RBPs and GEFT in CRC were examined through GEPIA database. Results showed that only LIN28A expression was positively associated with GEFT expression in CRC (**Supplementary Table 3**). Considering the vital roles of LIN28A in cancer progression, LIN28A was selected for further study. Next, the binding potential between LIN28A and GEFT mRNA was explored by RIP assay. Results showed that GEFT could be substantially enriched by LIN28A antibody in HCT-116 cells (**Figure 5B**). Prediction analysis revealed that LIN28A could bind with GGAGA motif in 3’UTR of GEFT mRNA (**Figure 5C**). Hence, RNA pull-down assay and luciferase assay were performed to further explore the binding possibility of LIN28A and GEFT 3’UTR. RNA pull-down assay coupled with western blot analysis showed that LIN28A could be significantly enriched by biotinylated sense GEFT 3’UTR, but not by biotinylated antisense GEFT 3’UTR in HCT-116 cells (**Figure 5D**). Luciferase assay showed that LIN28A overexpression remarkably enhanced the luciferase activity of wild type GEFT 3’UTR reporter, but not of mutant type GEFT 3’UTR reporter carrying mutant binding motif (*i.e.* ACGCG) (**Figure 5E**). These data showed that LIN28A could bind with GEFT 3’UTR via GGAGA motif. Next, RNA pull-down assay showed that LIN28A could be significantly enriched by biotinylated sense LINC00355, but not by biotinylated antisense LINC00355 in HCT-116 cells (**Figure 5F**). RIP assay also revealed that LINC00355 level were significantly increased in LIN28A immunoprecipitation complex than that in IgG immunoprecipitation complex in HCT-116 cells (**Figure 5G**), suggesting the binding activity of LIN28A protein and LINC00355. Moreover, we further demonstrated that LINC00355 knockdown led to the notable reduction of GEFT level enriched by LIN28A antibody in HCT-116 cells (**Figure 5H**). LINC00355 up-regulation promoted GEFT mRNA and protein expression and potentiated GEFT mRNA stability, while LIN28A knockdown markedly weakened the effects of LINC00355 on GEFT expression and mRNA stability in SW480 and HCT-116 cells (**Figures 5I, J**). These outcomes suggested that LINC00355 facilitated GEFT mRNA and protein expression and enhanced GEFT mRNA stability by LIN28A in CRC cells.

### LIN28A Knockdown Weakened the Promotive Effects of LINC00355 on Colorectal Cancer Cell Proliferation, Migration, and Invasion

**Figure 6:**
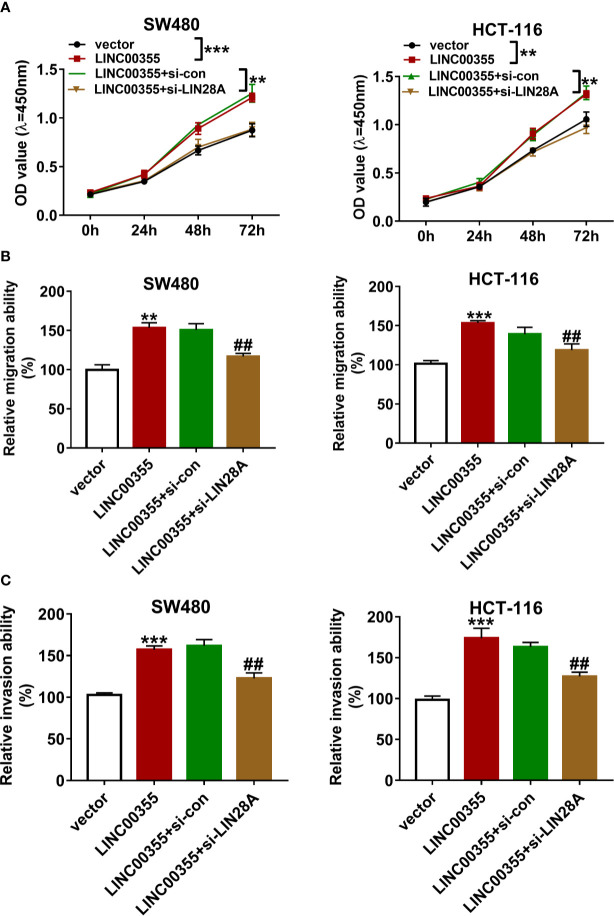
LIN28A knockdown weakened the promotive effects of LINC00355 on CRC cell proliferation, migration, and invasion. **(A–C)** SW480 and HCT-116 cells were transfected with empty vector, LINC00355 overexpression plasmid, LINC00355 overexpression plasmid + si-con, LINC00355 overexpression plasmid + si-LIN28A. **(A)** Cell proliferative ability was assessed by CCK-8 assay at the indicated time points post transfection. **(B, C)** At 24 h post transfection, cell migratory and invasive abilities were determined by Transwell migration and invasion assays, respectively. **P < 0.01. ***P < 0.001. ^##^ *P* < 0.01.

Next, we demonstrated that ectopic expression of LINC00355 markedly enhanced the proliferative, migratory, and invasive potential of SW480 and HCT-116 cells (**Figures 6A–C**). The depletion of LIN28A attenuated the promotive effects of LINC00355 on cell proliferation, migration, and invasion in SW480 and HCT-116 cells (**Figures 6A–C**).

## Discussion

In this project, we demonstrated that GEFT expression was markedly up-regulated in CRC tumor tissues compared to adjacent normal tissues, which was in line with our prior immunohistochemistry analysis (11). Moreover, our outcomes revealed that GEFT was highly expressed in several CRC cell lines (SW480, HCT-116 and HT-29) than that in a normal human colon epithelial cell line (FHC). Functional analysis presented that GEFT knockdown weakened CRC cell proliferative, migratory, and invasive capacities *in vitro* and suppressed CRC xenograft tumor growth *in vivo*. In view of the vital roles of GEFT in CRC tumorigenesis and progression and the close link of GEFT expression and CRC tumor metastasis or CRC patient prognosis, upstream regulatory mechanisms of GEFT were further investigated.

As mentioned above, lncRNA LINC00355 were picked out for further investigations by virtue of its overexpression in CRC tumor tissues and the negative association relationship between LINC00355 expression and CRC patient prognosis (32). LINC00355, also named as lnc-PCDH9-13:1, has been found to abnormally expressed in multiple malignancies such as esophageal squamous cell carcinoma (33), papillary renal cell carcinoma (34), and hepatocellular carcinoma (HCC) (35). Moreover, previous studies showed that LINC00355 was highly expressed and enforced expression of LINC00355 promoted the development and progression of cancers in HCC (35), lung adenocarcinoma (36), and head and neck squamous cell carcinoma (37). For instance, LINC00355 overexpression enhanced cell proliferative and migratory abilities in HCC (35). LINC00355 knockdown suppressed cell proliferation, facilitated cell apoptosis in lung adenocarcinoma cells, and hampered tumor growth in lung adenocarcinoma xenografts by reducing microRNA-195 expression and increasing cyclin E1 expression (36). Consistent with the previous report (32), we also demonstrated that LINC00355 expression was remarkably up-regulated in CRC tumor tissues *versus* normal tissues and CRC patients with high LINC00355 expression had a poor overall survival. Moreover, our data revealed that LINC00355 expression was positively associated with GEFT expression in CRC tumor tissues. LINC00355 positively regulated GEFT expression by improving GEFT mRNA stability in CRC cells. In addition, LINC00355 knockdown weakened CRC cell proliferative, migratory, and invasive capacities, while the detrimental effects of LINC00355 depletion on CRC cell proliferation, migration, and invasion were remarkably rescued by increased GEFT.

In view of the binding activity of RBPs and RNA transcripts including lncRNAs and mRNAs and regulatory roles of RBPs on gene expression, RBPs that could interact with both LINC00355 and GEFT were searched by Starbase database. Among common RBPs, LIN28A was selected in the light of its differential expression and association with GEFT in CRC. LIN28A has been found to be involved in the regulation of vital biological processes such as tissue development/repair, proliferation, invasion, metastasis, metabolism, and oncogenesis (38, 39). Previous study also showed that LIN28A could bind with some mRNAs to regulate mRNA splicing, translation, and stability (38, 40, 41). Moreover, LIN28A has been identified as an oncogene in multiple malignancies (38, 42). For instance, LIN28A loss weakened cell proliferative, migratory, and invasive abilities by increasing let-7a expression and reducing c-myc expression in papillary thyroid carcinoma (43). LIN28A promoted cell proliferation and invasion and suppressed cell apoptosis in ER−/Her2+ breast cancer cells and accelerated breast cancer xenograft tumor growth *in vivo* by activating androgen receptor (AR) *via* recruiting c-myc to AR promoter region (44). Additionally, LIN28A has been found to be highly expressed in CRC tumor tissues (45–47) and be a potential oncogenic gene in CRC (47, 48). For example, LIN28A facilitated tumor formation, growth, and invasion in Apc^Min/+^ CRC mice (47). LIN28A overexpression markedly improved the proliferative, migratory, and invasive capacities of CRC cells (49).

Our present study demonstrated that LIN28A could bind with LINC00355 or GEFT 3’UTR. LINC00355 up-regulation promoted GEFT expression and enhanced GEFT mRNA stability by LIN28A in CRC cells. Further functional analysis revealed that LIN28A loss weakened LINC00355-mediated pro-proliferation, pro-migration, and pro-invasion effects in CRC cells.

Taken together, our data revealed that GEFT exerted oncogenic effects in CRC and lncRNA LINC00355 worked in coordination with a RBP LIN28Ato regulate GEFT expression and function. Moreover, LINC00355overexpression markedly improved the proliferative, migratory, and invasive potential of CRC cells through increasing GEFT expression *via* LIN28A to 3’UTR region of GEFT. Our study suggested the potential diagnostic values of LINC00355 and GEFT in CRC and prognostic value of LINC00355 in CRC. Moreover, the elucidation of GEFT function and molecular regulatory mechanisms might contribute to the better management of CRC. The decryption of interactions between RBPs and their targets also can deepen our understanding on tumor biology and provide potential targets for cancer therapy.

## Data Availability Statement

The raw data supporting the conclusions of this article will be made available by the authors, without undue reservation.

## Ethics Statement

The studies involving human participants were reviewed and approved by the Research Ethics Committee of Affiliated Cancer Hospital of Zhengzhou University. The patients/participants provided their written informed consent to participate in this study. The animal study was reviewed and approved by the Animal Ethics Committee of Affiliated Cancer Hospital of Zhengzhou University.

## Author Contributions

YW designed and performed the experiments, and wrote the manuscript. BZ contributed to the experimental work and data analysis. GG and YZ conducted the experiments. QX revised the manuscript. All authors contributed to the article and approved the final version.

## Funding

This research was supported by the Medical Technology Research and Development Program of Henan Province (Grant nos. 201701029 and 182102310343).

## Conflict of Interest

The authors declare that the research was conducted in the absence of any commercial or financial relationships that could be construed as a potential conflict of interest.
