# Supplementary material for: Long Non-Coding RNA LINC00355 Promotes the Development and Progression of Colorectal Cancer by Elevating Guanine Nucleotide Exchange Factor T Expression via RNA Binding Protein lin-28 Homolog A
Source: Front Oncol. 2020 Dec 14;10:582669. doi: 10.3389/fonc.2020.582669 (PMC7769380; doi:10.3389/fonc.2020.582669)
Supplement: Supplementary file 1 [file DataSheet_1.docx]

Table 1 The upregualted lncRNAs (correlated with cancer processes) in colorectal cancer samples by TCGA analysis.

| gene_name | gene_type | baseMean | log2FoldChange | lfcSE | p_value | p_adj |
| --- | --- | --- | --- | --- | --- | --- |
| FEZF1-AS1 | lncRNA | 161.4248 | 9.320184 | 0.469943 | 1.56E-87 | 8.56E-85 |
| LINC01234 | lncRNA | 133.6307 | 7.744789 | 0.471442 | 1.21E-60 | 1.91E-58 |
| LINC02163 | lncRNA | 21.54541 | 7.244914 | 0.399341 | 1.48E-73 | 4.92E-71 |
| LINC00460 | lncRNA | 63.60408 | 7.108677 | 0.409844 | 2.16E-67 | 4.88E-65 |
| LINC00659 | lncRNA | 47.63923 | 6.255824 | 0.350878 | 4.2E-71 | 1.15E-68 |
| LINC00858 | lncRNA | 52.1028 | 6.172425 | 0.371237 | 4.47E-62 | 7.74E-60 |
| AFAP1-AS1 | lncRNA | 405.747 | 5.863363 | 0.471203 | 1.52E-35 | 5.33E-34 |
| FIRRE | lncRNA | 56.58592 | 5.773948 | 0.341099 | 2.82E-64 | 5.43E-62 |
| LINC01614 | lncRNA | 18.33448 | 5.679444 | 0.443633 | 1.6E-37 | 6.44E-36 |
| MIR31HG | lncRNA | 6.903934 | 5.394807 | 0.533453 | 4.84E-24 | 8.11E-23 |
| LINC01050 | lncRNA | 5.897461 | 5.368492 | 0.46988 | 3.13E-30 | 7.86E-29 |
| LINC01511 | lncRNA | 8.439043 | 5.117284 | 0.502956 | 2.58E-24 | 4.4E-23 |
| LINC02428 | lncRNA | 5.254864 | 5.06722 | 0.637754 | 1.94E-15 | 1.7E-14 |
| ELFN1-AS1 | lncRNA | 279 | 4.932669 | 0.22868 | 3.4E-103 | 3E-100 |
| LINC00355 | lncRNA | 4.544516 | 4.928991 | 0.837918 | 4.04E-09 | 1.91E-08 |
| CASC19 | lncRNA | 45.12648 | 4.897391 | 0.269215 | 6.04E-74 | 2.02E-71 |
| AC007128.1 | lncRNA | 32.42407 | 4.895039 | 0.275538 | 1.31E-70 | 3.53E-68 |
| LINC02223 | lncRNA | 5.087489 | 4.881347 | 0.474565 | 8.15E-25 | 1.45E-23 |
| BBOX1-AS1 | lncRNA | 90.21288 | 4.866649 | 0.270721 | 2.97E-72 | 8.8E-70 |
| LINC01996 | lncRNA | 12.08014 | 4.844521 | 0.442784 | 7.33E-28 | 1.59E-26 |

Table 2 The differentially expressed RBPs in colorectal cancer samples among 44 common RBPs by TCGA analysis.

| gene_name | baseMean | log2FoldChange | lfcSE | p_value | p_adj |
| --- | --- | --- | --- | --- | --- |
| LIN28A | 4.03648 | 3.512895 | 0.678166 | 2.22E-07 | 8.51E-07 |
| LIN28B | 2.981886 | 3.457939 | 1.055168 | 0.001049 | 0.0023 |
| IGF2BP3 | 155.8786 | 4.180483 | 0.388049 | 4.61E-27 | 9.5E-26 |
| IGF2BP1 | 127.3965 | 4.4544 | 0.444286 | 1.17E-23 | 1.89E-22 |
| NOP56 | 5415.81 | 1.408024 | 0.099676 | 2.62E-45 | 1.73E-43 |
| FBL | 8059.657 | 1.098881 | 0.115589 | 1.97E-21 | 2.72E-20 |
| NOP58 | 3149.262 | 1.23044 | 0.087458 | 5.89E-45 | 3.82E-43 |

Table 3 The correlation of differentially expressed RBPs and GEFT

| RBP_name | R_value | p_value |
| --- | --- | --- |
| LIN28A | 0.23 | 1.30E-04 |
| LIN28B | 0.094 | 1.20E-01 |
| IGF2BP3 | 0.048 | 4.30E-01 |
| IGF2BP1 | 0.034 | 5.70E-01 |
| NOP56 | -0.098 | 1.00E-01 |
| FBL | -0.18 | 2.10E-03 |
| NOP58 | -0.18 | 3.40E-03 |
